# Lycopene suppresses palmitic acid-induced brain oxidative stress, hyperactivity of some neuro-signalling enzymes, and inflammation in female Wistar rat

**DOI:** 10.1038/s41598-021-94518-5

**Published:** 2021-07-22

**Authors:** Regina Ngozi Ugbaja, Adewale Segun James, Emmanuel Ifeanyichukwu Ugwor, Adio Jamiu Akamo, Funmilola Clara Thomas, Ayokulehin Muse Kosoko

**Affiliations:** 1 Department of Biochemistry, College of Biosciences, Federal University of Agriculture, P.M.B 2240, Abeokuta, Ogun State Nigeria; 2 Department of Chemical Sciences (Biochemistry Program), Augustine University Ilara-Epe, Epe, Lagos State Nigeria; 3 Department of Veterinary Physiology and Pharmacology, College of Veterinary Medicine, Federal University of Agriculture, P.M.B 2240, Abeokuta, Ogun State Nigeria; 4 Department of Pharmacy, School of Applied Sciences, University of Huddersfield, Queensgate, HD1 3DH UK

**Keywords:** Biochemistry, Neuroscience

## Abstract

Neuroinflammation can be triggered by certain high caloric nutrients such as palmitic acid (PA). The effect of lycopene against PA-induced neuroinflammation in female rats has not been as explored. In the present study, thirty rats (weighing 150–200) g were randomly allotted into six groups (n = 5) comprising normal control, PA control, PA + lycopene (0.24 mg/kg), PA + lycopene (0.48 mg/kg), lycopene (0.24 mg/kg), and lycopene (0.48 mg/kg), respectively. After seven weeks of PA challenge (5 mM) including two weeks of lycopene treatment, the brain was excised for analyses. Palmitic acid overload caused significant (p < 0.05) increases in adenosine deaminase, monoamine oxidase-A, nucleotides tri-phosphatase, 5′-nucleotidase, acetylcholine esterase, and myeloperoxidase activities, and malondialdehyde (MDA) levels which were reduced significantly in the lycopene-treated groups. Conversely, catalase and glutathione peroxidase activities, and reduced glutathione levels concentration decreased by 43%, 34%, and 12%, respectively in the PA control groups compared with the Control. Also, PA triggered a decrease in the brain phospholipids (11.43%) and cholesterol (11.11%), but increased triacylglycerol level (50%). Furthermore, upregulated expressions of Interleukin-1β, Interleukin-6, and NF-ĸB-p65 in the PA control were attenuated, while decreased Interleukine-10 expression was upregulated due to lycopene treatment. Severe brain vacuolation observed in the histology of the PA control rats was normalized by lycopene. This study concludes that lycopene ameliorated PA-induced neuroinflammation, probably via attenuation of oxidative stress, and downregulation of TLR4/ NF-κB -p65 axis.

## Introduction

Neuro-inflammation (due to meta-inflammation) describes an immuno-metabolic disorder of the nervous system, arising from derangements of the metabolic and inflammatory pathways in response to various cues including infection, autoimmunity, or certain high caloric nutrients^1^. Nutrients (such as free fatty acids) may accumulate in the nervous tissues such as the microglia due to excessive positive energy balance. The sustained impaired energy balance may invoke glial cells activation and disproportionate production of reactive oxygen species (ROS). Moreover, dysregulation of lipids, such as triacylglycerol and cholesterol correlates positively with neurodegenerative diseases^2^. Either the excessive generation of ROS or accumulated free fatty acids, such as palmitic acid (PA) might activate the canonical inflammatory pathways, and elicit chronic inflammation of the brain^3^. The chronic inflammation may later degenerate into a myriad of neuro-psychopathologies, including major depressive disorder, Parkinson’s, and Alzheimer’s diseases^1^.

Excessive intake of PA, a long-chain saturated fatty acid, and an agonist of the immune cells -modulating toll-like receptor-4 (TLR-4), might orchestrate the downstream cross-talks among many adaptor proteins such as myeloid differentiation primary response 88 (Myd88), consequently activating the nuclear factor kappa B (NF-κB) via the MyDD8/TRAF/IKK pathways^4^. NF-κB activation often induces the hyper-proliferation of pro-inflammatory cytokines such as interleukin-1beta (IL-1β), and interleukin-6 (IL-6), and downregulate the anti-inflammatory phenotypes such as interleukin-10 (IL-10)^5^. The hyper-proliferation and infiltration of these pro-inflammatory mediators and high metabolic demand of the brain predispose it to oxidative stress, which exacerbates the neuroinflammation^6^. Also, neuropathic events are usually characterized by disturbances of neuro-behavioural enzymes such as acetylcholine esterase, monoamine oxidase- A, and adenosine deaminase^7,8^. Understanding the aetiology of neuro-inflammation from the energy homeostasis perspective is important to the long-term prevention of neuro-pathologies. More so, pharmacological intervention remains a therapeutic possibility in neuropathic management^3^.

Lycopene (C_40_H_56_), an aliphatic polyunsaturated carotene-containing compound, has been classified as a functional food and is abundant in tomatoes, watermelons, papaya, and guava^2^. Believed to be one of the most potent anti-inflammatory phytochemicals, it also abrogates oxidative stress (OS) via chain-breaking mechanisms and electron donation in vivo. It has been shown that lycopene has modulatory activities against various neurodegenerative diseases^9^. As experimental evidence suggests, female are more susceptible to inflammation-related diseases relative to their male counterparts worldwide^10,11^. Furthermore, a careful review of literature also shows that females are more prone to neuroinflammatory disorders, such as Alzheimer’s disease (AD), amyotrophic lateral sclerosis (ALS), and depression^12^. The above reasons informed the choice of female animals used in this study, while the dearth of information regarding PA-induced neuroinflammation in female rats warrants this study. This study, therefore, aimed to investigate the possible therapeutic effects of lycopene on PA-induced OS, disruption of neuro-behavioural enzymes and inflammatory indices in female Wistar rats.

## Results

### Lycopene abolishes derangements in brain neuro-behavioural enzymes activities

**Figure 1:**
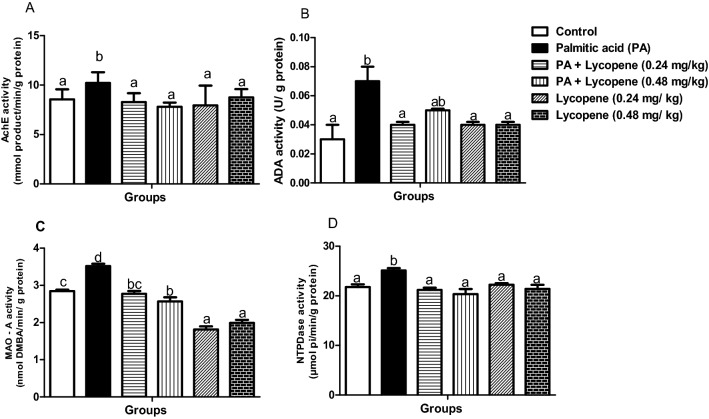
Effects of lycopene on the activities of neuro-behavioural marker enzymes of palmitic acid-induced neuro-inflammation in female rats. Values are expressed as mean ± S.E.M (n = 5). Bars with different letters are statistically distinct (p < 0.05). (**A**) AchE – acetylcholine esterase (**B**) ADA- adenosine deaminase (**C**) MAO-A- monoamine oxidase- A, (D) NTPDase – nucleotide triphosphatidase. Data were analysed using SPSS version 20 (IBM SPSS Software, United Kingdom) while the graph was plotted using GraphPad Prism Software, version 6 (San Diego, CA 92,108) https://www.graphpad.com/scientific-software/prism/.

Palmitic acid (PA) caused a significant (p < 0.05) increase in the brain AchE activity (19.28%) when compared with the control group (Fig. 1A). Nevertheless, the activity was abated in groups treated with 0.48 mg/kg lycopene (23.60%) relative to the PA control group. ADA activity (Fig. 1B) increased dramatically (by 133.3%) in the PA–treated group. The same trends were observed, for the activities of MAO-A (23.59%) and NTPDase (15.35%) Regardless, 0.48 mg/kg of lycopene significantly lowered the activities of MAO-A (26.78%) (Fig. 1C) and NTPDase (18.96%) (Fig. 1D) as well as ADA (28.57%). There was no significant difference between the groups treated with lycopene alone (0.24 mg/kg and 0.48 mg/kg) (Fig. 1), and the Normal control.

### Lycopene abated palmitic acid-induced oxidative stress in rat brain

**Figure 2:**
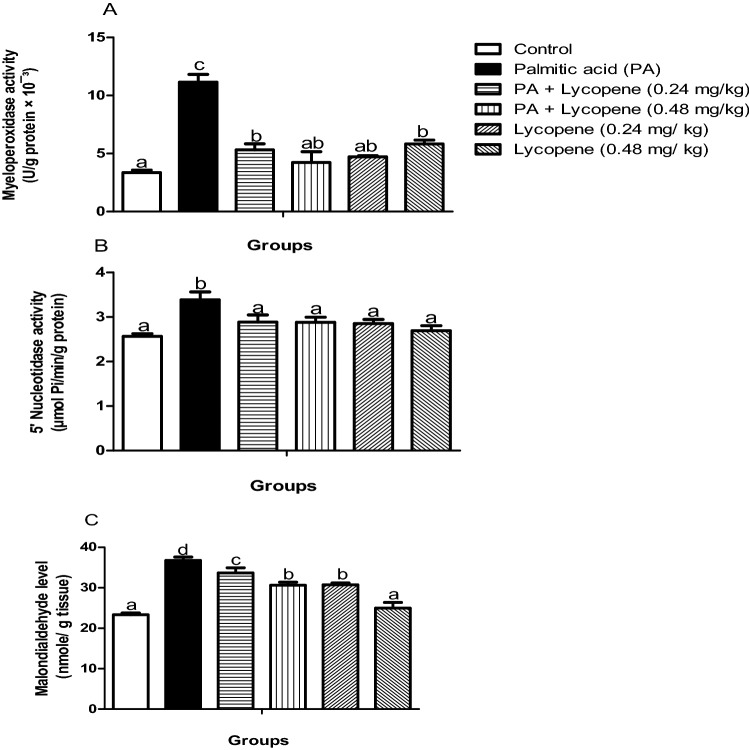
Effects of lycopene on oxidative stress markers of palmitic acid-induced neuro-inflammation in female rats. Values are expressed as mean ± S.E.M (n = 5). Bars with different letters are statistically distinct (p < 0.05). (**A**) Myeloperoxidase activity (**B**) 5′ Nucleotidases activity (**C**) MDA level. Data were analysed using SPSS version 20 (IBM SPSS Software, United Kingdom) while the graph was plotted using GraphPad Prism Software, version 6 (San Diego, CA 92,108) https://www.graphpad.com/scientific-software/prism/.

Following two weeks of intervention with lycopene, the increased activities of 5-NTD (15.35%) and MPO (232.84%) of PA- challenged animals reduced significantly (p < 0.05) by 18.96% and 62%, respectively by 0.48 mg/kg lycopene. Similarly, the MDA level increased (57.63%) significantly (p < 0.05) in the PA control group when compared with normal control, which was abated by lycopene (0.48 mg/kg). There was no significant difference in the 5-NTD and MPO activities between the two lycopene only dose (0.24- and 0.48 mg/kg) compared to the control group (Fig. 2). The MDA level decreased more in the group treated with 0.48 mg/kg of lycopene when compared with the 0.24 mg/kg lycopene group.

### Lycopene improves antioxidant markers in rats challenged with palmitic acid (PA)

**Figure 3:**
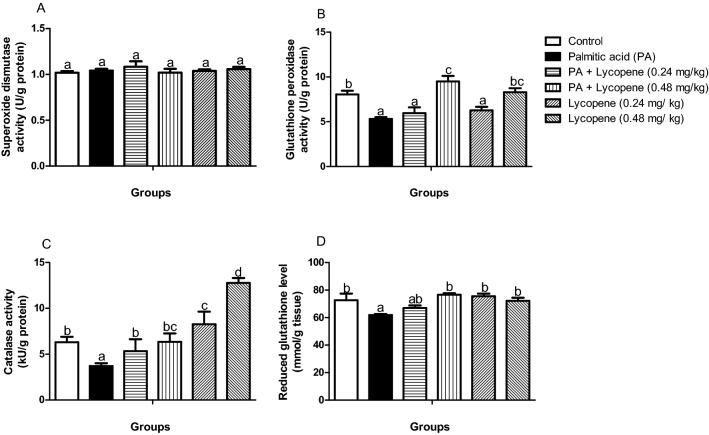
Effects of lycopene on the activities of antioxidant markers of palmitic acid-induced neuro-inflammation in female rats. Values are expressed as mean ± S.E.M (n = 5). Bars with different letters are statistically distinct (p < 0.05). (**A**) Superoxide dismutase activity (**B**) Glutathione peroxidase activity (**C**) Catalase activity (**D**) Reduced glutathione level. Data were analysed using SPSS version 20 (IBM SPSS Software, United Kingdom) while the graph was plotted using GraphPad Prism Software, version 6 (San Diego, CA 92,108) https://www.graphpad.com/scientific-software/prism/.

There was no significant difference (p > 0.05) in the SOD activity (Fig. 3A) across the groups. The brain activities of GPx, CAT, and GSH level decreased significantly (p < 0.05), by 43%, 34%, and 12%, correspondingly in the palmitic acid PA- challenged group when compared with the control group (Fig. 3). Lycopene-supplemented groups showed a remarkable increase in the downregulated enzymes in a dose-dependent manner. Commendably, the group challenged with PA but treated [PA + lycopene (0.48 mg/kg)] showed more improved activities of GPx (Fig. 3B) and CAT (Fig. 3C) relative to the normal control group. However, there was no significant difference in GSH level (Fig. 3D) between the PA + lycopene (0.48 mg/kg) group, and normal control as is SOD activity (Fig. 3A).

### Palmitic acid (PA)—mediated derangement of lipid metabolism in the rat brain was remediated by lycopene

**Figure 4:**
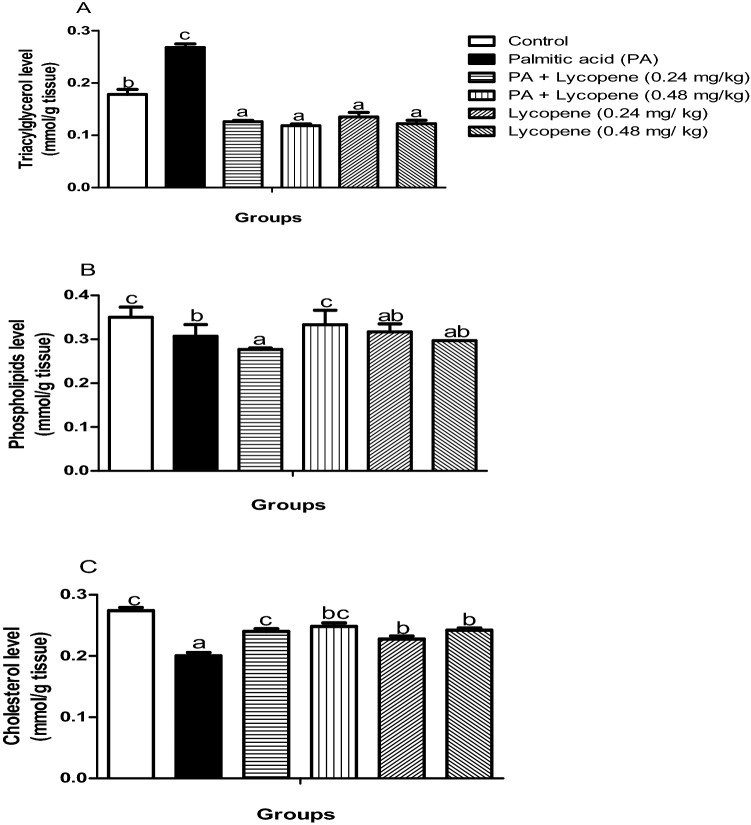
Effects of lycopene on brain lipid profiles of palmitic acid-induced neuro-inflammation in female rats. Values are expressed as mean ± S.E.M (n = 5). Bars with different letters are statistically distinct (p < 0.05). (**A**) Triacylglycerol level (**B**) Phospholipids level (**C**) Cholesterol level. Data were analysed using SPSS version 20 (IBM SPSS Software, United Kingdom) while the graph was plotted using GraphPad Prism Software, version 6 (San Diego, CA 92,108) https://www.graphpad.com/scientific-software/prism/.

Phospholipids (Fig. 4B) and cholesterol (Fig. 4C) levels were significantly (p < 0.05) reduced by 11.43% and 11.11% respectively in the PA-exposed rats, while triacylglycerol (Fig. 4A) by (50%) increased slightly when compared with the control group. Regardless of the perturbations observed in the PA control group, lycopene intervention reversed the disturbances in all the lycopene treated groups. There was no significant difference in the level of TAG (Fig. 4B) among the entire lycopene-treated group.

### Lycopene modulated the induction of brain inflammatory cytokines in palmitic acid-exposed rats

**Figure 5:**
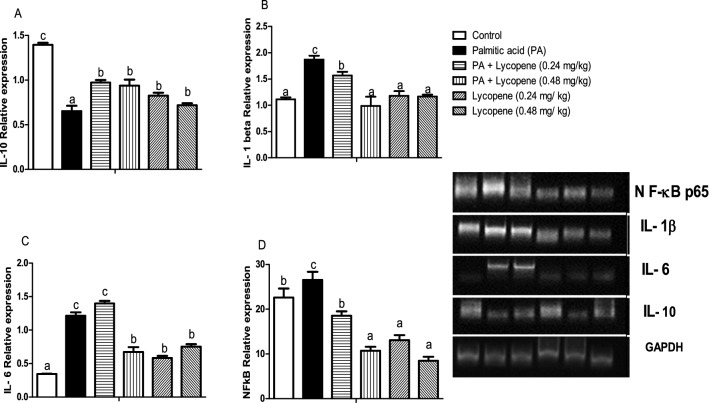
Effects of lycopene on the inflammatory gene expression levels of palmitic acid-induced neuro-inflammation in female rats. Values are expressed as mean ± S.E.M (n = 5). Bars with different letters are statistically distinct (p < 0.05). (**A**) Interleukin- 10 level (**B**) Interleukin 1- beta level (**C**) Interleukin -6 level (**D**) NF-kB level. Data were analysed using SPSS version 20 (IBM SPSS Software, United Kingdom) while the graph was plotted using GraphPad Prism Software, version 6 (San Diego, CA 92,108). https://www.graphpad.com/scientific-software/prism. The agarose gel intensity was quantified using Image J, Version 8. (https://github.com/imagej/imagej1).

Hyper-activation of brain NF-ĸB-p65 is a hallmark of the palmitic acid PA group (Fig. 5D) when compared with the normal control group. The relative expression of IL-1β and IL-6 similarly increased in the PA-untreated group, while IL-10 relative expression significantly decreased in the PA-untreated group. Lycopene supplementation significantly (p < 0.05) upregulated the expression of IL-10 (Fig. 5A), downregulated IL-1β (Fig. 5B), IL-6 (Fig. 5C), and NF-ĸB-p65 (Fig. 5D). The downregulation occurred in a dose-dependent manner. There was no significant difference between the two doses lycopene only groups (0.24- and 0.48 mg/kg) (Fig. 5).

### Lycopene improves histo-morphological alterations of rats challenged with palmitic acid.

**Figure 6:**
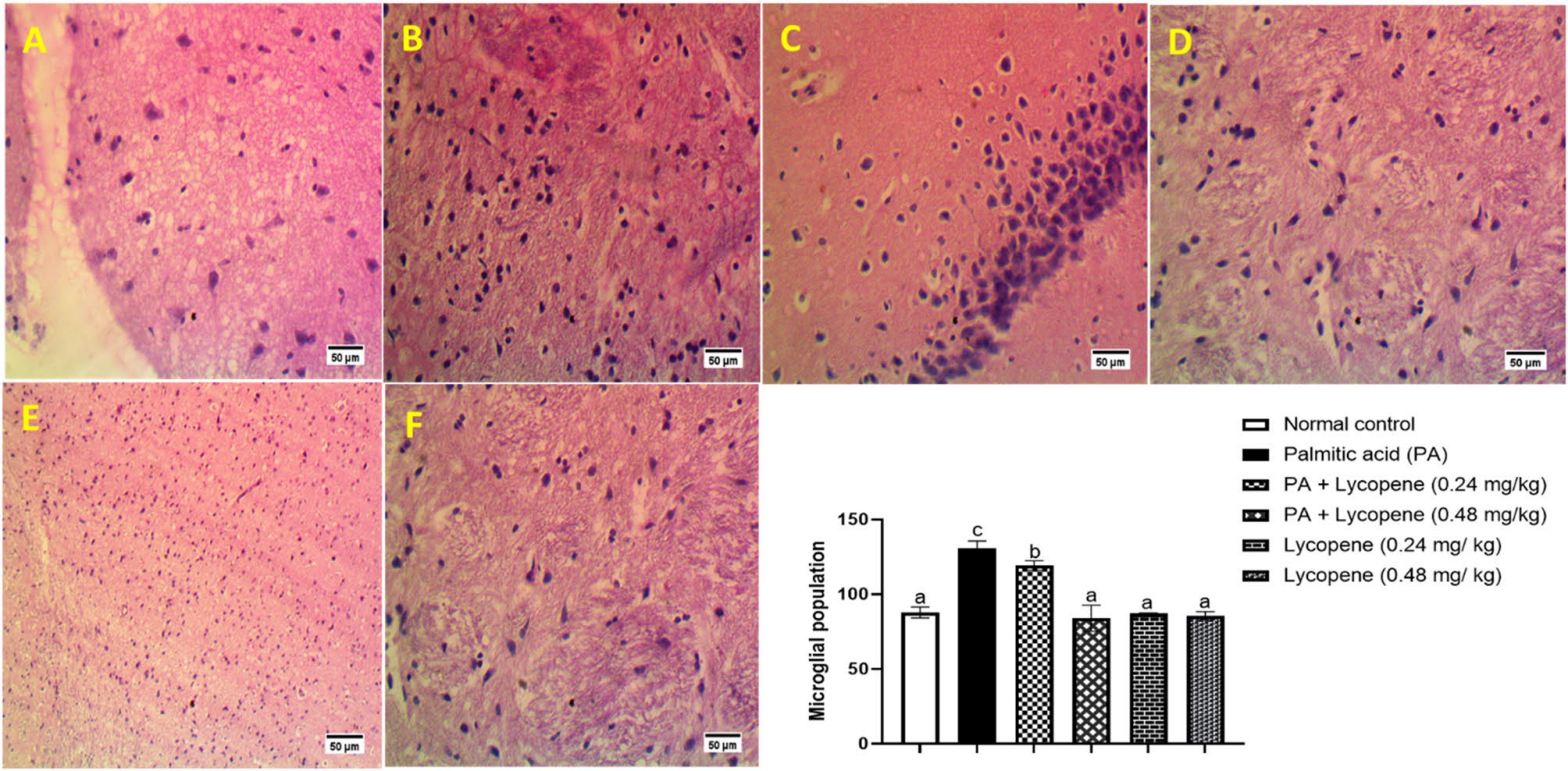
Photomicrograph of rat brain submitted to palmitic acid and treated with lycopene (Hematoxylin and Eosin staining × 400μ; Scale = 50μm). A. Normal control shows moderate vacuolation of the brain tissue (arrowhead). B. Shows PA control with severe vacuolation and a high number of microglia cells population. C. PA + lycopene (0.24 mg/kg) shows normal architecture. D. PA + lycopene (0.48 mg/kg) shows brain tissue with normal architecture (arrow head). E. Lycopene (0.24 mg/kg) group shows normal histology. F. Lycopene (0.48 mg/kg) shows normal histology.

In Fig. 6, histological evaluation of the brains shows that palmitic acid PA caused severe vacuolation when compared with the normal control, where there was moderate vacuolation. The histology of the groups treated with lycopene appeared to improve the brain architecture apart from groups that were not treated with PA but treated with lycopene which showed mild vacuolation.

## Discussion

In this study, PA overload caused the elevation of acetylcholine esterase (AchE), adenosine deaminase (ADA), monoamine oxidase- A (MAO-A), and nucleotide triphosphatase (NTPDase) activities. Acetylcholine esterase is an enzyme involved in the breakdown of acetylcholine to choline and acetate at the post-synaptic junctions of the central nervous system^8^. Hyperactivity of AchE depletes acetylcholine and is prodromal to neurodegenerative disease and type 2 diabetes (T2D)^13^. Elevation of AchE activity in this study might be due to systemic low-grade inflammation resulting from depletion of acetylcholine; a neurotransmitter known for its anti-inflammatory activity^14^. Indeed, acetylcholine is capable of inhibiting the NF-kB activation and production of pro-inflammatory mediators such as TNF-α, and IL-1β^15^. Elevated AchE activity following PA overload suggests the ability of PA overload to initiate neuroinflammation. To our knowledge, this study is the first to report the implicative role of PA overload in cholinergic signalling disruption in the rat. We observed that lycopene had an inhibitory effect on acetylcholine esterase activity in the treated groups. The modulation of AchE activity by lycopene was previously reported following BPA-induced intoxication in rats^16^. Similarly, lycopene was shown to ameliorate Scopolamine-induced amnesia by lowering the activity of AchE in mice^17^. The neuroprotective effect of lycopene might be due to its ability to permeate the blood–brain- barrier and ROS scavenging^18^, or attenuation of OS^17,19^.

ADA is a mammalian purine-metabolizing enzyme that metabolizes adenosine-a regulatory metabolite with anti-inflammatory function^20^. ADA acts by converting adenosine to inosine, via the removal of the amino group of the adenosine^21^. The intracellular level of adenosine is strictly regulated by, and linked to energy status in the tissue^20^. In this context, the catabolic role of ADA might influence its immunomodulatory effects^20,22^. Although, the implication of ADA in neurodegeneration remains unclear, however, activation of the T- cells by adenosine suggests that aberrant catabolism of adenosine by ADA might induce inflammation. The concentration of PA used in our study (5 mM) exceeds the physiological PA level (0.5 mM)^15^, suggesting that, while the body metabolizes the required PA; the excess accumulates in the brain leading to surplus calories. The excessive calorie then increases the brain adenosine level that necessitates increased catabolism by ADA^20^. Avci and Durak^7^ suggested that increased ADA activity correlates positively with pathological conditions linked to a deranged immune system and inflammation. Also, to our knowledge, we are reporting for the first time, the involvement of ADA in PA-induced neuroinflammation in rats. Nevertheless, the activity of ADA was normalized in the lycopene supplemented group, further affirming the therapeutic effect of lycopene against neuro-inflammatory triggers and mediators^23^. Considering the putative role of ADA in inflammatory diseases, the anti-inflammatory effects of lycopene might be responsible for the observed reduction of ADA activity in the lycopene-treated groups^7^.

Akin to the aforementioned enzymes, MAO-A disruption has been implicated in neurologic disorders^24^. Monoamine oxidases are a group of flavin-containing enzymes that cleaves the amino group from biogenic and xenobiotic amines in vivo. Their function includes mood control, motivation, and reward in the brain^13^. Experimental evidence has linked MAO-A hyperactivity to increased ROS-induced inflammation^25^. More tellingly, increased MAO-A activity in the brain raises the brain ammonia level, and accumulation of ammonia in the brain might cause ATP depletion^26^. Thus, the elevated activity of MAO-A in this study suggests that habitual consumption of PA- rich diet might trigger a cascade of cognition deficit-inducing condition via OS and inflammation induction. We however observed a meaningful reduction of MAO-A activity in the lycopene-supplemented groups. Our observation is consistent with that of El-Morsy and Ahmed^16^, who suggested that the ability of lycopene to diminish cognitive deficit might be due to attenuation OS and upregulation of BDNF. Alternatively, the anti-inflammatory effect of lycopene might be responsible for the lowering of MAO-A in the rat brain.

The NTPDases activity usually shows the extent of nucleotide breakdown in the cell (ATP hydrolysis) and might be used as an index for the bioenergetic status of the brain^24^. ATPase breaks down ATP into ADP and Pi with a concomitant release of energy for the metabolic process, while 5′-Nucleotidase (5′-NTD) breaks down AMP to adenosine and Pi. The cumulative effect of these enzymatic reactions is a wholesale reduction in brain nucleotide pools and energy levels^27^. In our study, NTPDase and 5′-NTD activities increased significantly in the PA-treated groups suggests PA-mediated reduction in brain nucleotide pool, energy shortage, impaired purinergic signalling, necrosis, and possible cell death^28^. Furthermore, the generation of ROS due to excessive PA metabolism might induce brain mitochondrial dysfunction and uncouple energy generation thereby causing neural apoptosis and cognition impairment^29^. Our data showed that lycopene reversed these purinergic enzymes abnormalities suggesting an increase in ATP levels necessary for proper brain functioning. These data portend beneficial attributes possessed by lycopene in the management of impaired neuro-behavioural enzymes due to PA intoxication. Our observation is consistent with the study of Malekiyan et al*.*^30^, where lycopene attenuated neuronal damages induced by diabetes in rats by improving the purinergic enzyme activities.

The causal role of OS in neurodegenerative disorders is undisputable. Perhaps, due to the large oxidative-species-generating capacity of the brain, its limited antioxidative ability, and abundance of polyunsaturated fatty acids, the brain cells are susceptible to oxidative damage^31^. Oxidative stress is driven by ROS when the cells' ability to neutralize them is overwhelmed, or when the antioxidant system (including enzymes such as catalase, and glutathione peroxidase) is impaired^32^. PA overload causes over-activity of the respiratory chain and increases ROS production via Complexes III and IV, as well as uncoupling of ATP synthesis^29^. Our data shows that PA causes OS by significantly lowering the catalase and glutathione peroxidase activities (Fig. 3). PA also caused a copious increase in the MDA level (an index of lipid peroxidation) coupled with abatement in GSH level in the brain of the untreated PA-challenged rats. The inhibition of catalase and glutathione peroxidase specific activities, following the PA challenge is consistent with the observations of Alnahdi et al.^33^ who reported that high PA and glucose caused a significant reduction of the antioxidant enzymes and caused upregulation of inflammatory cytokines. Further, the activity of MPO was evaluated in the brain to substantiate other sources of ROS and inflammation following PA administration. Myeloperoxidase is produced by polymorphonuclear cells with pro-inflammatory and pro-oxidative functions. It is secreted by activated monocytes, microglia, and macrophages as a direct response to oxidative stress. It also increases the production of pro-inflammatory cytokines^34,35^. The MPO in conjugation with hydrogen peroxide might cause the elevation of MDA and activate inflammation via the up-regulation of inducible nitric oxide synthase (iNOS)^34,36^. Upregulation of MPO following the PA challenge in this study, lends more credence to its ability to elicit cellular stress when taken in excess. Nevertheless, lycopene administration essentially abated the occurrence of OS by normalizing the activities of CAT, GPx, and MPO as well as augmenting the level of GSH in the treated groups. Accordingly, the MDA level decreased meaningfully in the lycopene-treated rats when compared with the untreated group. Consistent with earlier reports on the attenuation of OS indices in the hippocampus of diabetic rats^30^, our observations further substantiate the antioxidant effect of lycopene and its ability to inhibit lipid peroxidation, break the chain of ROS production, and induce antioxidant enzymes^16,30^.

Excessive PA has putative detrimental effects on whole-body metabolism, especially *de novo* lipogenesis^37^. Disproportionate energy balance might disrupt the tightly controlled regulation of tissue PA, thus leading to dyslipidemia, hyperglycemia, and ectopic fat accumulation, and activate the inflammatory TLR4/NF-ĸB-p65 pathways^37^. Indeed, disruption of PA homeostatic balance has been implicated in atherosclerosis and neurodegenerative disease^37,38^. We measured some brain lipids levels to ascertain if PA could disrupt its metabolism as part of /or an alternative mechanism of PA-mediated neuroinflammation. Our data revealed a significant reduction in the brain phospholipids and cholesterol contents with a concomitant increase in TAG level in the PA untreated group (Fig. 4). This trend suggests that PA might cause an imbalance in lipid metabolism in the brain. The mechanism behind these observations is unclear. However, de novo lipogenesis (DNL) and exogenous PA are reciprocally regulated to maintain a balanced saturated fatty acid/unsaturated fatty acid (SAFA/UFA) ratio. Excessive PA is desaturated and incorporated into the cell membrane. The desaturation process is necessary to maintain the concentration of phospholipids at the physiological range^38^. However, increased PA level distorts the SAFA/UFA ratio in favour of SAFA, causing a reduction in the phospholipids contents^39^. This disturbance might be responsible for the lowering of phospholipids levels observed in this study. Furthermore, an imbalance of SAFA/UFA might influence the transcription factors responsible for cholesterol and TAG biosynthesis. The net result is disruption of TAG and cholesterol production. As observed in this study, TAG level increased in the PA-untreated group, and TAG elevation is prodromal to inflammatory responses^37^. Lycopene has been shown to possess the ability to reduce intracellular lipids owning to its hypolipidemic effect^40^. Cholesterol level decreased in the PA control group relative to control in our study. The underlying mechanism behind this observation is unclear. However, reduction of cholesterol level might limit the level of precursor for estrogen 2 (E2)-a hormone produced by females, which is known to possess an anti-inflammatory effect by inhibiting the proliferation of TNF-α, IL-1β, and IL-6, while enhancing the synthesis of IL-10^41,42^. This observation might be responsible for the inflammatory responses exhibited by the PA group and might be another mechanism behind greater susceptibility to metabolic inflammation in females^42^. Nevertheless, treatment with lycopene reversed the PA-induced reduction in cholesterol level.

Neuroinflammation is driven by the master regulator of inflammation—NF-κB^43^ via TLR4, which culminates into the secretion of pro-inflammatory cytokines such as IL-1β, IL-6, and TNF-α^5^. In this study, upregulation of the NF-κB -p65, parallels with concomitant increments in the expression of gene targets such as IL-1β, and IL-6 (Fig. 5). This suggests that PA-induced the activation of the TLR4/MyD88/NF-kB signalling cascade leading to the production of the pro-inflammatory cytokines. Our observations are consistent with other studies^33,44^, wherein PA caused the upregulation of pro-inflammatory mediators. Besides, upregulation of IL-1β in the CNS during HFD feeding might contribute to neuroinflammation and blood–brain-barrier (BBB) disruption^45^. IL-10 relative expression, on the other hand, decrease in the PA control group, suggesting that PA overload might decrease the expression of anti-inflammatory cytokines, which may aggravate neuronal damage^46^. Nevertheless, our results showed normalization of IL-10 level in the lycopene-treated groups suggesting its ability to enhance anti-inflammatory cytokine production. Regardless of perturbations imposed on the inflammatory pathways by PA, lycopene essentially and abated inflammatory response in the groups treated with lycopene, especially the 0.48 mg/kg dose. This observation did not only authenticate the anti-inflammatory effect of lycopene but its neuroprotective effects^23,30^, perhaps due to its lipophilicity and ability to easily cross the blood–brain barrier to modulate cellular processes.

Disruption of biochemical processes often results in histological impairments and this represents the basis for tissue damage and loss of function^47^. In the present study, the brain of animals in the PA group showed severe vacuolation and excessive microglial population (Fig. 6). Vacuolation in the brain cytoplasm is a degenerative feature arising from xenobiotic insult^47^ and is mostly caused by damaged myelin sheath in the brain, suggesting that PA could initiate a complex yet unresolved process that induces demyelination of the CNS. Groups treated with lycopene, however showed normal brain architecture when compared with the untreated group, thereby substantiating the neuroprotective role of lycopene against PA-induced neuroinflammation.

## Conclusion

**Figure 7:**
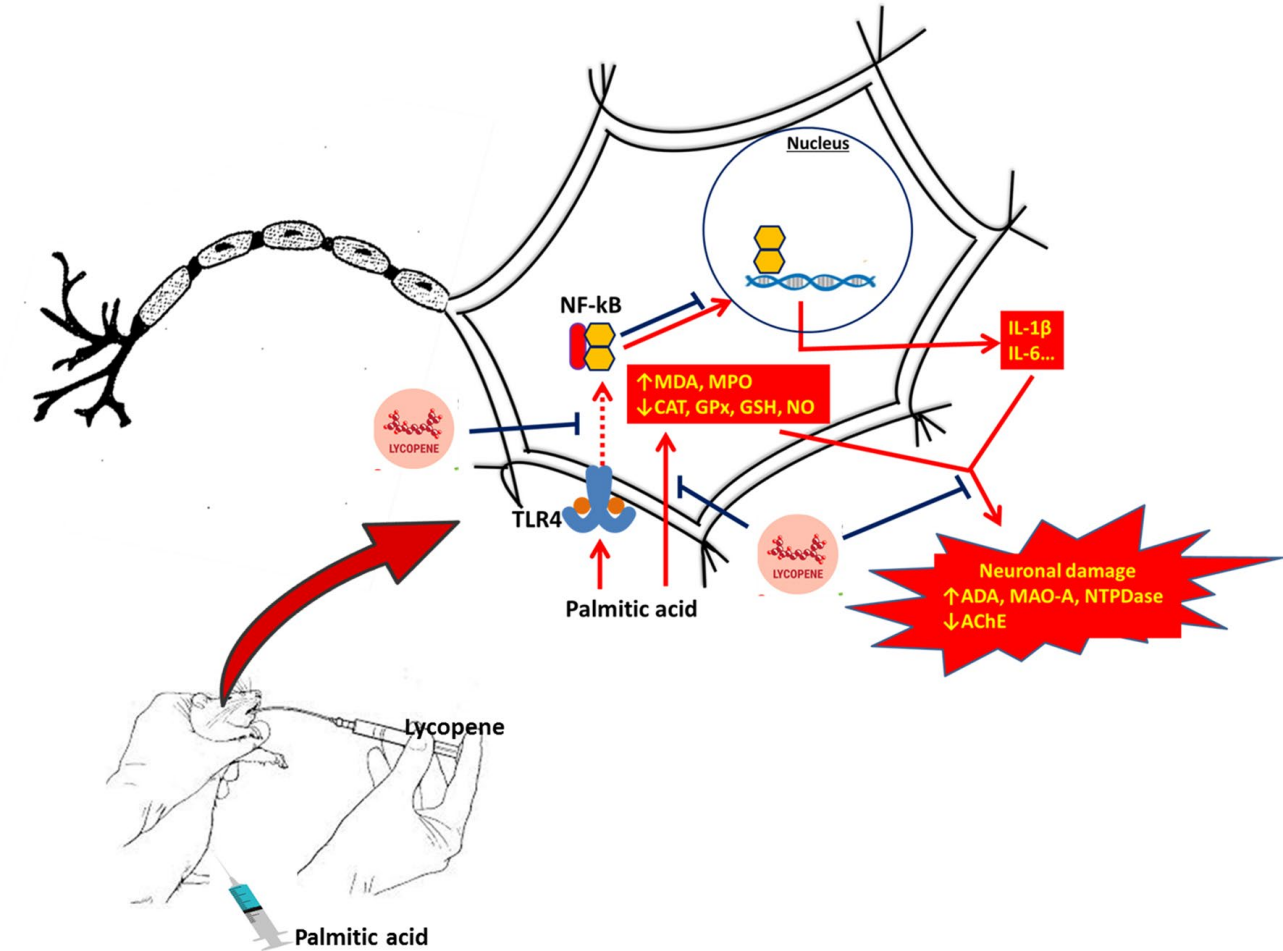
Graphical abstract.

Conclusively, excessive PA intake caused the alteration of neuro-behavioural enzymes activities and induced OS by inhibiting the antioxidant system. Furthermore, it might also cause dysregulation of lipid metabolism, as well as upregulation of the mRNA levels of pro-inflammatory cytokines. Nevertheless, lycopene showed tremendous ameliorative effects of PA-induced neuroinflammation in rats. This is characterized by normalization of neurobehavioral enzymes, attenuation of OS indices, modulation of lipid metabolism, and abatement of inflammatory cytokines. The underlying mechanism might be through the attenuation of OS and downregulation of TLR4/NF-κB -p65 inflammatory axis. The conclusion is summarized as a graphics in Fig. 7.

## Materials and methods

### Chemicals and reagents

Lycopene (All-*Trans*) and TRIzol were purchased from Solarbio Life Science and Co. Ltd. Beijing, China. PA, fatty acid–free bovine serum albumin (BSA), Guaiacol, adenosine triphosphate (ATP), acetylthiocholine iodide, adenosine, 5′-5′-dithiobis- (2-dinitrobenzoic acid) (DTNB) and Pyrogallol were obtained from Sigma Aldrich (St. Louis, Missouri, United States). All other chemicals used were of pure and analytical grade. The relative expressions of mRNA coding for IL-1β, IL-6, IL-10, NF-κB -p65 and GAPDH were quantified using the primer sequences synthesized by ShineGene Bio-Technologies, *Inc*. Shanghai, Xuhui District (China). Total cholesterol, triacylglycerol and phospholipids kits were sourced from Labkits® Diagnostics Company (Barcelona, Spain).

### Methods

#### Experimental animals

Thirty (30) female Wistar rats (8 weeks of age) were purchased and kept in the Animal Housing Unit of the Department of Biochemistry, Federal University of Agriculture, Abeokuta (FUNAAB), Ogun State, Nigeria. The animals were acclimatized for two weeks before the commencement of the experiment. They were housed in plastic cages with good ventilation and supplied with standard pellets and clean water ad *libitum*. All animals were handled humanely according to the guidelines for use of experimental animals, and the study conformed to the Animal Research: Reports of In vivo Experiments (ARRIVE) guidelines for use of experimental animals^48^. Ethical approval (FUNABCH170641) was obtained for the study from the Ethical Committee of the Department of Biochemistry, FUNAAB.

#### Induction of neuroinflammation and experimental design

Neuroinflammation was induced via administration of 5 mM PA intraperitoneally (i.p.) for five weeks. PA was complexed with 1% Bovine Serum Albumin (BSA) and given five times weekly at 100 µL/100 g body weight. The ratio of PA to BSA was 8:1^49^. Briefly, 50 mM PA (stock solution) was initially dissolved in 50% ethanol and incubated at 60º C and later brought to 5 mM using the 1% BSA (dissolved at 35 $^\circ$C)^50^. The resulting PA-BSA complex was kept at −20 $^\circ$C and thawed when needed. After the first five weeks, animals were grouped and treated as shown in Table 1 (without halting PA administration together with the lycopene treatment), for two weeks. The total study duration was for seven weeks. The lycopene was reconstituted in Olive oil and administered to the animal via oral gavage. The dose of lycopene chosen was based on a previous study^51^, which reported a dose of 7–21 mg/70 kg/ day to be beneficial in humans, so the equivalent dose of 0.24 mg/kg body weight of the rats, and a double dose of 0.48 mg/kg were used in this study.

**Table 1:** Experimental design.

Groups	First 5 weeks*	Treatment(last 2 weeks)
Normal control	1% BSA	+ vehicle
PA	5 mM PA + 1% BSA	+ vehicle
PA + Lycopene (0.24 mg/kg)	5 mM PA + 1% BSA	+ lycopene (0.24 mg/kg)
PA + Lycopene (0.48 mg/kg)	5 mM PA + 1% BSA	+ lycopene (0.48 mg/kg)
Lycopene (0.28 mg/ kg)	1% BSA	+ lycopene (0.24 mg/kg)
Lycopene (0.48 mg/kg)	1% BSA	+ lycopene (0.48 mg/kg)

*The respective PA and/or BSA administration lasted throughout the study; BSA = Bovine serum albumin; PA = palmitic acid; vehicle = olive oil.

#### Animal sacrifice and tissue preparation

After the end of seven weeks, the animals were fasted overnight and sacrificed by cervical dislocation following anaesthesia using ketamine/xylazine (35-/5 mg/kg). Brain tissue was excised, blotted dry, and preserved at −20 °C before biochemical analyses.

#### Preservation of tissues for histology and gene expression analyses

A section of brain tissue was fixed in 10% formalin for histological assessment^52^, while a small portion of the brain was preserved in 100 µL of TRIZOL reagent for gene expression analyses. The gene expression samples were kept at −20 °C until analysed.

### Biochemical analyses

#### Assay for neuro-behavioural marker enzymes

The Acetylcholine esterase (AchE) activity was determined as briefly described. A working reagent (2 mL total volume), containing 0.1 M potassium phosphate buffer, pH 7.5, and 1 mmol DTNB was incubated with the homogenate. The method is based on the formation of the yellow anion- 5, 5-dithiol-bis-acid-nitrobenzoic, measured by absorbance at 412 nm for 3 min. The reaction was initiated by the addition of 0.8 mM acetylthiocholine iodide to the sample in the working reagent. The enzyme activity was expressed as nmol AChE/min/mg protein^27^.

Monoamine oxidase-A (MAO-A) activity was estimated using benzyl-amine as the substrate^27^. Briefly, the reaction mixture 250 µL (containing 0.1 M phosphate buffer, pH 7.4, 200 µM benzyl-amine, and 40 µL of the sample) was incubated for 1 h at 37 °C and later cooled on ice. Then, 500 µL of distilled water, 250 µL of 10% zinc sulphate, and 50 µL of 1 M NaOH were added, incubated for 2 min on the ice, and centrifuged (10,000 g for 10 min). Finally, the supernatant was diluted five times with 1 M NaOH, and the absorbance read at 450 nm.

Adenosine deaminase (ADA) activity was assayed, as a direct measurement of the formation of ammonia produced when adenosine deaminase reacts with an excess of adenosine^24^. Concisely, 50 µL of the sample was reacted with 21 mM of buffered adenosine; pH 6.5, incubated for 1 h at 37 °C. 300 µL of phosphate buffer was added to 200 µL of 0.15 mmol/L of ammonium sulphate solution. The mixture was incubated for 30 min at 37 $^\circ$C followed by the addition of 1 mL of phenol/ sodium nitroprusside (106/0.17 mmol/L) to the tubes to terminate the reaction. Then 1 mL of 125 mmol/L NaOH was added to the tubes followed by incubation at 37 $^\circ$C for 30 min, and the absorbance monitored at 630 nm.

Nucleotide triphosphatase (NTPDase) activity was determined in the brain homogenate by NTPDase enzymatic assay as described by Schetinger et al.^53^. A reaction medium (containing 5 mM KCl, 1.5 mM CaCl_2_, 0.1 mM EDTA, 10 mM glucose, 225 mM sucrose and 45 mM Tris–HCl buffer, pH 8.0), in a final volume of 200 µL was prepared. 20 µL of the homogenate was added to the reaction mixture and pre-incubated at 37 $^\circ$C for 10 min. The reaction was initiated by the addition of 10 µL of ATP (1.0 mM) and incubation allowed to proceed for 20 min. The reaction was terminated by adding 100 µL of 5% sodium dodecyl sulfate (SDS) and the amount of inorganic phosphate liberated quantified as described by Katewa and Katyare^54^. Briefly, 750 µL of diluted 1.25% (w/v) ammonium molybdate [10 µL of 2.5% ammonium molybdate (prepared with 3 N H_2_SO_4_) in 65 µL of 1.062 N H_2_SO_4_] was added to the mixture, followed by 100 µL of reducing agent (20 mg each of hydralazine sulphate and ascorbate in 1 ml of 0.1 N H_2_SO_4_). The whole reaction mixture was thoroughly mixed, and the absorbance measured at 820 nm against blank within 30 min. The activity of NTPDase in the sample was extrapolated from the phosphate standard curve.

5′ nucleosidases (5-NTD) activity was estimated as per the method of El-Asser and El- Merzabani^55^. Briefly, 70 μL of assay buffer (containing 62.5 mM Tris/HCl pH 7.4 and 62.5 mM MgCl_2_) and 10 μL of freshly prepared adenosine monophosphate (AMP) and 100 mM L-cysteine (10 μL) was pipetted into well-labelled Eppendorf tubes and allowed to equilibrate at 37 °C for 5 min. Afterwards, samples (10 μL) was added, vortexed thoroughly and incubated at 37 °C for 45 min. The reaction was terminated by adding 5% SDS (50 µL) and the amount of inorganic phosphate (Pi) liberated quantified as described by Katewa and Katyare^54^ The activity was indirectly calculated as the amount Pi liberated per minute.

#### Assay for antioxidant and oxidative stress markers

The superoxide dismutase (SOD) activity was based on the inhibition of the auto-oxidation of pyrogallol according to Marklund and Marklund^56^. Briefly, 20 µL of the sample was allowed to incubate with 180µL of 100 mM Tris–HCl buffer, pH 8.2 for 10 min. Then, 50 µL of 10 mM pyrogallol was added to initiate the reaction, monitored for 3 min at 420 nm.

Catalase (CAT) activity was assayed for as described by Shangari and O’Brien^57^, based on the yellow complex formation between the excess hydrogen peroxide and ammonium molybdate at physiological pH. Briefly, 0.1 ml of the sample was incubated with 1 mL of buffered hydrogen peroxide (0.1 M phosphate buffer, pH 7.4 and 65 µL/mL), after 1 min, the reaction was terminated with 32.4 mM of ammonium molybdate, and the absorbance read at 405 nm.

Glutathione peroxidase (GPx) activity was assayed based on the conjugation of 5′-5′-dithiobis- (2-dinitrobenzoic acid) (DTNB) with the excess thiol group present after the sample has been precipitated with 10% TCA^58^. In short, 25 µL of the homogenate was added to 75 µL GPx working reagent (containing 4 mM GSH, 10 mM NaN3, 2.5 mM H2o2, and 0.1 M phosphate buffer, pH 7.4) and allowed to stand for 10 min. Later, 25µL of 10% TCA was used to terminate the reaction and centrifuged at 3500 rpm for 10 min. 35 µL of the supernatant was reacted with 350 µL of GSH working reagent (containing 10 mM DTNB and 0.3 M Tris- HCl buffer containing 1 mM EDTA). The absorbance was taken 10 min later at 412 nm.

Myeloperoxidase (MPO) activity was assayed for in the post-nuclear fraction of the brain tissue as described by Klebanoff et al.^59^ with slight modifications. Briefly, 10µL of the homogenate was reacted with 10 µL of 4 M guaiacol and 100 mM H_2_O_2_ in 0.1 M phosphate buffer, pH 7.0 and the change in absorbance were monitored for a minute at 470 nm. The lipid peroxidation (MDA) level was determined by measuring the formation of thiobarbituric acid reactive substances (TBARS), according to the method of Buege and Aust^60^. 0.5 ml of the sample (distilled water for blank) was treated with 1.0 ml of TBA reagent (containing 0.35% TBA, 25 mM HCl, and 15% TCA) and was incubated in boiling water bath for 15 min. The tube was immediately placed on ice to cool, centrifuged at 3500 rpm for 10 min and the absorbance of clear supernatant measured against blank at 532 nm.

Reduced glutathione (GSH) level was assayed according to Ellman^61^ method. Briefly, 25 µL of the sample was precipitated using 10% TCA. Then, the homogenate was centrifuged at 3500 rpm for 10 min. 35 µL of the supernatant was then reacted with 350 µL of GSH working reagent (containing 10 mM DTNB and 0.3 M Tris- HCl buffer containing 1 mM EDTA), the absorbance read at 412 nm.

#### Brain lipids level determination

Brain triacylglycerol (TAG), cholesterol (CHOL), and phospholipids (PHOL) were evaluated after the lipid was extracted from the brain tissue as described previously^62^ using the commercial test kits. Briefly, 0.2 g of the brain was homogenized in chloroform/methanol mixture (2:1) and centrifuged at 3000 rpm for 10 min. The chloroform layer (500 µL) was then transferred into another Eppendorf tube, dried on a water bath at 60 °C. Triton-X (10 µL) was added to the extract to precipitate any protein and dried again. The lipid content (TAG, CHOL, and PHOL) was then estimated with the test kits according to the manuals.

### Gene expression analyses of the inflammatory markers

Brain total mRNA was extracted in the TRIzol, The mRNA was converted to cDNA, and amplified using EasyScript one-time RT PCR Supermix (Cat No: AE411), produced by TransGen Biotech Co. (Beijing, China). Afterwards, the relative expression of genes coding for interleukin 1-beta (IL-1β), interleukin 6 (IL-6), interleukin 10 (IL-10), and NF-κB were quantified following agarose gel electrophoresis using Image J software, version 8 (https://github.com/imagej/imagej1)^63^. The relative expression was normalised against GAPDH. Primer sequences for the target genes are shown in Table 2.

**Table 2:** Gene target primer sequences.

Gene Target	Forward sequences	Reverse sequences
NF-κB-p65	TCCCACAAGGGGACATTAAGC	CAATGGCCTCTGTGTAGCCC
IL-1β	CCTTTGGCAAGTGTCTGAAGC	TCAGACAGCACGAGGCATTT
IL-6	TCCGGAGAGGAGACTTCACA	GAATTGCCATTGCACAACTCTT
IL-10	TGCGACGCTGTCATCGATTT	GTAGATGCCGGGTGGTTCAA
GAPDH	AGTGCCAGCCTCGTCTCATA	GATGGTGATGGGTTTCCCGT

### Histological evaluation of the brain

A small portion of the brain tissue fixed in 10% formalin was cut (about 3 µm) and stained with Hematoxylin and Eosin, and later viewed under a microscope^64^.

### Statistical analysis

Values are expressed as mean ± standard error mean (SEM). Data were analysed by One-way Analysis of variance (ANOVA) followed by the Duncan Multiple Range Test (DMRT) with p < 0.05 significance level. All these analyses were done using Statistical Package for Social Sciences (SPSS) version 20 (IBM SPSS Software, United Kingdom) The band densities of the gene expression analyses were quantified using Image-J, while all the graphs were plotted using Graph Pad Prism version 6 (San Diego, CA 92,108).

## Supplementary Information

Supplementary Information.
