# Supplementary material for: Lycopene suppresses palmitic acid-induced brain oxidative stress, hyperactivity of some neuro-signalling enzymes, and inflammation in female Wistar rat
Source: Sci Rep. 2021 Jul 22;11:15038. doi: 10.1038/s41598-021-94518-5 (PMC8298469; doi:10.1038/s41598-021-94518-5)
Supplement: Supplementary file 1 — Supplementary Information. [file 41598_2021_94518_MOESM1_ESM.docx]

**Lycopene Suppresses Palmitic acid-induced Brain Oxidative Stress, Hyperactivity of Some Neuro-signalling Enzymes, and Inflammation in Female Wistar rat**

Regina Ngozi Ugbaja^a,b^*, Adewale Segun James^a^, Emmanuel Ifeanyichukwu Ugwor^a^, Adio Jamiu Akamo^a^ , Funmilola Clara Thomas^c^, Ayokulehin Muse Kosoko^a^,^d^

Affiliations: ^a^ Department of Biochemistry, College of Biosciences, Federal University of

Agriculture, Abeokuta, Ogun State, Nigeria P.M.B 2240

^b^ Department of Chemical Sciences (Biochemistry Program), Augustine University Ilara-Epe, Lagos State Nigeria

^c^ Department of Veterinary Physiology and Pharmacology, College of Veterinary Medicine, Federal University of Agriculture, Abeokuta, Ogun State, Nigeria. P.M.B 2240

^d^ Department of Pharmacy, School of Applied Sciences, University of Huddersfield, Queensgate, HD1 3DH United Kingdom

Correspondence: R. N. Ugbaja

Email address: ugbajarn@funaab.edu.ng;

regina.ugbaja@augustineuniversity@edu.ng

Telephone: +2348066050043.

Gel Bands for the genes


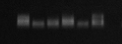
 Brain IL-10


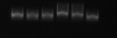
 Brain Gapdh


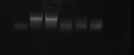
 Brain IL-6


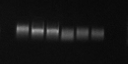
 Brain IL-1Beta


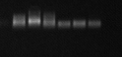
 Brain NF-kB

Brain NFkB (2)


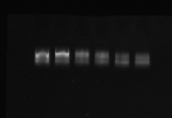


Brain Gapdh (2)


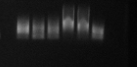


Brain IL -10 (2)


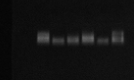


Brain IL-6 (2)


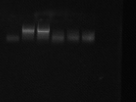


Brain IL-1Beta (2)


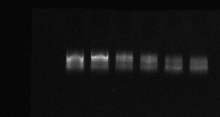


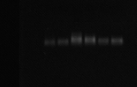
 Brain IL-10 (3)


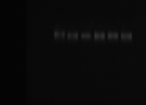
 Brain Gapdh (3)


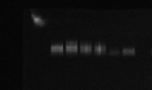
 Brain IL-6 (3)


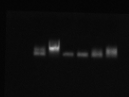
 Brain NF-kB (3)


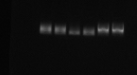
Brain IL-1 beta (3)
